# Copper-Modified Mesoporous Silica Nanoparticles for Antimicrobial Applications

**DOI:** 10.3390/nano15241884

**Published:** 2025-12-15

**Authors:** Amaia M. Goitandia, Maialen Argaiz, Miren Blanco, Giorgia Grilli, Elisa Recchia, Alessandra Amoroso, Nathalie Totaro, Andrea Ciammaruconi, Riccardo De Santis, Leire Ruiz Rubio, Fabiana Arduini, Florigio Lista

**Affiliations:** 1 Unidad de Química de Superficies y Nanotecnología, Fundación Tekniker, Iñaki Goenaga 5, 20600 Eibar, Spain; amaia.martinez@tekniker.es (A.M.G.); maialen.argaiz@tekniker.es (M.A.); 2 Defence Institute for Biomedical Sciences, 00184 Rome, Italy; giorgia.grilli@persociv.difesa.it (G.G.); elisa.recchia@persociv.difesa.it (E.R.); alessandra.amoroso@persociv.difesa.it (A.A.); nathalie.totaro@persociv.difesa.it (N.T.); andrea.ciammaruconi@persociv.difesa.it (A.C.); r.desantis@uniroma1.it (R.D.S.); florigio.lista@esercito.difesa.it (F.L.); 3 Department of Public Health and Infectious Diseases, Sapienza University of Rome, Piazzale Aldo Moro 5, 00185 Rome, Italy; 4 Macromolecular Chemistry Group (LQM), Physical Chemistry Department, Faculty of Science and Technology, University of the Basque Country (UPV/EHU), 48940 Leioa, Spain; leire.ruiz@ehu.eus; 5 Department of Chemical Science and Technologies, University of Rome “Tor Vergata”, Via Della Ricerca Scientifica 1, 00133 Rome, Italy; fabiana.arduini@uniroma2.it

**Keywords:** mesoporous silica nanoparticles (MSNs), antibacterial properties, virucidal properties, virus inactivation, SARS-CoV-2, influenza A, *E. coli*, *S. aureus*

## Abstract

The escalating global crisis of antimicrobial-resistant (AMR) bacterial infections, along with the continuous threat of viral outbreaks, poses a serious risk to public health worldwide and underscores the urgent need for innovative therapeutic strategies. In this study, mesoporous silica nanoparticles (MSNs) were successfully synthesized and subsequently functionalized with copper to impart broad-spectrum antimicrobial activity. The oxidation state of copper on the MSN surface was modulated through thermal treatments, allowing the evaluation of its influence on antimicrobial efficacy. The modified MSNs were tested against key bacterial pathogens, including *Escherichia coli* and *Staphylococcus aureus*, achieving complete bactericidal activity after 2 h of exposure to *E. coli.* Moreover, as well as influenza A (H1N1) pdm09, severe acute respiratory syndrome coronavirus 2 (SARS-CoV-2) and MS2 bacteriophage (MS2) were evaluated, reaching an efficiency higher than 80%, 90%, and 97%, respectively. The results indicated that copper-modified MSNs exhibit potent antibacterial and antiviral activity, highlighting their potential as an antibiotic-free alternative for preventing microbial infections while mitigating the development of AMR bacteria.

## 1. Introduction

Antimicrobial resistance is a critical global health issue. It occurs when microorganisms, such as bacteria, viruses, and fungi, develop the ability to resist the effects of medications designed to combat them, such as antibiotics, antivirals, antifungals, and antiparasitic. This makes it difficult to treat infections and increases the risk of disease spread, severe and prolonged illness, and increased morbidity and mortality rates [1,2]. In particular, the growth of biofilm confers substantial tolerance to killing, while subpopulations of dormant persister cells further reduce bactericidal efficacy [3].

It is estimated that bacterial AMR was directly responsible for 1.27 million global deaths in 2019 and contributed to 4.95 million deaths [4,5]. In addition to death and disability, AMR has significant economic costs. The World Bank estimates that AMR could result in US$1 trillion additional healthcare costs by 2050, and US$1 trillion to US$3.4 trillion gross domestic product (GDP) losses per year by 2030 [6]. Moreover, viral infections are threatening the health of human beings. In particular, coronavirus disease 2019 (COVID-19) caused by the severe acute respiratory syndrome coronavirus 2 (SARS-CoV-2) infection has claimed nearly 6.9 million lives worldwide by May 2023 due to deadly pneumonia since the outbreak at the end of 2019 [7]. Consequently, there is a pressing need to develop novel approaches to address this emergency, highlighting the urgent requirement for alternative and more effective antimicrobial agents.

In this context, nanotechnology has achieved substantial advances in medicine, therapeutics, drug delivery, and biotechnology over the last two decades [8]. In particular, inorganic nanoparticles (NPs) possess enhanced efficacy compared to conventional antibiotics, owing to their small sizes and large surface area [9]. In addition, inorganic NPs are also gradually moving to the clinical stage, with about 25 inorganic nanomedicines approved for clinical use [10]. Among them, mesoporous silica nanostructures have acquired great scientific importance. The synthesis of the MSN is rather feasible even at a large kilogram scale, and it is based on sol–gel chemistry, which involves two main steps: the hydrolysis and condensation of silica precursors around a template generated via the supramolecular self-assembly of surfactants in an aqueous solution, followed by the elimination of the template by using calcination or solvent extraction [11,12,13]. Moreover, they presented outstanding properties, such as a large specific surface area (800–100 m^2^/g), tunable pore size (2–5 nm), and particle size (typically <200 nm), high mechanical, thermal, and chemical stability, and easy internal/external surface functionalization, as well as a superior safety profile compared to other inorganic nanoparticles [14,15,16].

These characteristics have facilitated their use to avoid the generation of highly resistant bacteria. Although MSNs have no antimicrobial properties by themselves, the functionalization, adsorption, or loading with different antimicrobials allows for the preparation of materials with enhanced antimicrobial properties [11,17]. Adsorbed or functionalized antimicrobials are usually not delivered, and their effectiveness is related to an effective rise in the local concentration of the antimicrobial due to their anchoring or adsorption on the MSN surface [18,19,20]. Moreover, MSNs can also serve as suitable containers for antimicrobials, both commercial antibiotics [21,22] or other biocides [23,24], that are delivered by a simple diffusion from the mesopores to the medium, or, in case of smart-polymer-functionalized particles, owing to a predefined stimulus, such as temperature, pH changes, enzymes, certain chemical species, or light, thus ultimately achieving a specific administration and action [25,26,27]. However, despite the obvious advantage of using this approach, it has not been fully explored, and further, simpler-to-implement advances in this area are needed [11]. A further advantage of using MSNs is its potential for supporting metallic antibacterial compounds, in the form of metal nanoparticles or metal ions. These systems leverage the intrinsic antimicrobial properties of metals to disrupt bacterial membranes, generate reactive oxygen species (ROS), and interfere with metabolic pathways. Additionally, they address several limitations of traditional antibiotics, including resistance and the ability to penetrate biofilms.

Among metallic agents [28,29,30,31,32,33], silver has been extensively studied for MSN-based antimicrobial systems [28,29], whereas copper remains underexplored [34]. Copper emerges as a promising antimicrobial alternative, as it is the only metal with antimicrobial properties that is also an essential micronutrient for humans and all other living things including cell respiration, neurotransmitter synthesis, and the crosslinking of collagen and elastin in the skin. This means that, while it can be toxic in high concentrations, it is generally less problematic than other metals, such as silver [35]. Copper is antimicrobial across all temperatures and all levels of humidity, whereas silver requires temperatures of 35 °C and 95% RH. In addition, the average price for silver is around $400/pound while the average price for copper is around $3.50/pound, less than 1% the price of silver [36]. Reports on copper-modified MSNs exist but remain scarce and often lack the systematic evaluation of synthesis parameters and their impact on antimicrobial performance [37,38]. Existing studies typically focus on antibacterial activity, with limited attention to antiviral properties or the role of copper oxidation states [12].

This study addresses these gaps by (i) optimizing MSN synthesis for a high surface area and tunable porosity, (ii) incorporating copper under controlled conditions to modulate its oxidation state, and (iii) evaluating both the antibacterial and antiviral efficacy, including for SARS-CoV-2, influenza A, and MS2 bacteriophage. To our knowledge, this is the first systematic study linking copper speciation on MSNs to dual antibacterial and virucidal properties, providing a foundation for antibiotic-free strategies to mitigate AMR and viral transmission.

Specifically, in the study, the state of the copper on the surface was modified by using thermal changes, to examine the influence of this copper state on the efficacy of the nanoparticles against pathogenic bacteria, specifically *Staphylococcus aureus* (Gram-positive) and *Escherichia coli* (Gram-negative). These two groups are different in their cell wall structures, which respond differently to antimicrobial compounds. Gram-negative bacteria, compared to Gram-positive bacteria, have a more complex cell wall, which makes these bacteria more resistant to antibiotics, particularly those that target the cell wall [3]. *S. aureus* and *E. coli* are common agents that can cause infections in humans and animals. *S. aureus* is a clinically relevant organism that can cause various diseases, mainly bloodstream infections, pneumonia, and skin infections, especially in individuals with chronic illnesses [39], while *E. coli* is a commonly studied bacterium that can be found in the environment and in the gastrointestinal tract of humans and animals [40]. Most of the *E. coli* strains are commensal, which contribute to the normal functioning of the intestinal microbiota, whereas pathogenic strains expressing specific virulence factors can cause gastrointestinal disease like bloody diarrhea. These bacteria are widely studied and used in the laboratory for their easy cultivation, simple genetic manipulation, and long-standing use as model organisms in microbiology research [39,40], as well as their role as reference organisms for antimicrobial testing according to ISO 22196 [41]. Moreover, they are used to investigate virulence factors, bacterial growth, and antimicrobial resistance mechanisms. In addition, the following viruses, namely, the influenza A (H1N1) pdm09 virus and the severe acute respiratory syndrome coronavirus 2 (SARS-CoV-2), were selected based on their clinical and epidemiological significance [42]. The ease of transmission of respiratory viruses through both the inhalation of aerosols and droplets from infectious individuals and fomites, as well as contaminated surfaces, makes these viruses a global threat to public health and the economy worldwide. The impact of the SARS-CoV-2 pandemic on healthcare and economic systems, along with the countermeasures implemented, is a fine case in point for the gravity of these events. Moreover, MS2 bacteriophage (MS2) has been selected as a reference model for non-enveloped viruses, generally more resistant to chemical disinfectants and environmental stress factors due to the lack of a lipid envelope and, therefore, more difficult to inactivate [43].

## 2. Materials and Methods

### 2.1. Materials

**Figure 1:**
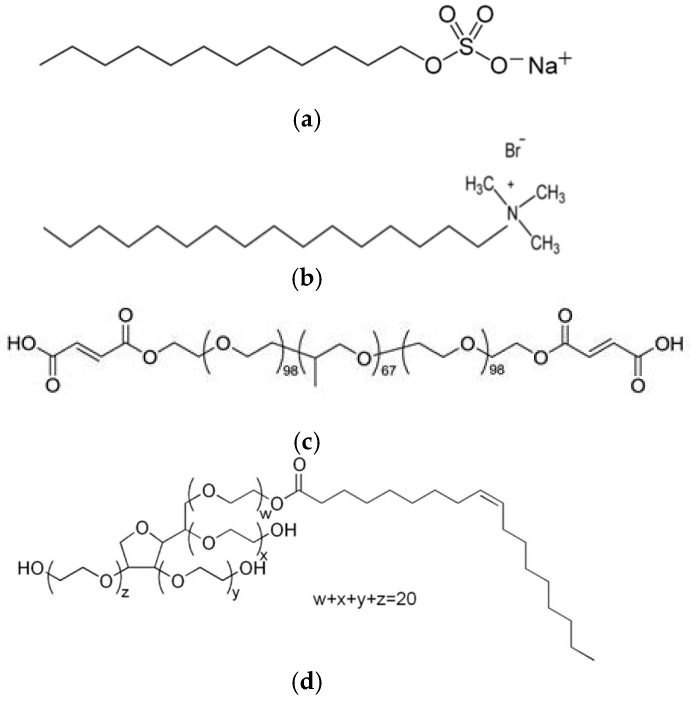
Images of the chemical structure of (**a**) SDBS; (**b**) CTAB; (**c**) Pluronic F 127; and (**d**) Tween 80.

For the synthesis of silica mesoporous nanostructures, tetraethylorthosilicate (TEOS, 98%) supplied by Thermo Scientific (Waltham, MA, USA) was employed as precursor. Deionized water (DI) obtained from a Millipore Elix (Merck Millipore, Billerica, MA, USA), and ethanol (99.8%, HPLC grade) from Scharlab (Barcelona, Spain) were employed in the synthesis. As catalyst, ammonium hydroxide (28 wt.%), ethyl (methyl)amine (MEA), and triethylamine (TEA), all from Scharlab, were evaluated. Different types of template molecules or surfactants were employed in the synthesis: two ionic surfactants, sodium dodecylbenzenesulfonate (SDBS) and N-Cetyl Trimethyl Ammonium Bromide (CTAB), and two non-ionic surfactants, Pluronic F127 and Polysorbate 80 (Tween 80). All of them were supplied by Sigma-Aldrich (St. Louis, MO, USA) and their chemical structure was shown in Figure 1. Finally, different pore controllers were explored, hexane, heptane, dodecane, cyclohexane, and mesitylene, from Sigma-Aldrich. The source of copper was copper sulphate Cu_2_SO_4_, also from Scharlab.

### 2.2. Synthesis of Cu-Modified MSNs

For the synthesis of the silica mesoporous nanoparticles, firstly, the surfactant was dissolved in a mixture of ethanol, H_2_O, and the catalyst, maintaining the mixture under stirring for 5 min until the complete dissolution of the surfactant. If needed in the synthesis, pore expander was added to the mixture, and the mixture was maintained under stirring for 15 min. When using mesitylene as pore expander, as the mixture was not homogeneous, an additional step was incorporated in the process, consisting of homogenizing the mixture for 15 min at 10,000 rpm by using a Ultraturrax IKA T25 (IKA‑Werke GmbH & Co. KG, Baden-Württemberg, Germany). Subsequently, TEOS was added dropwise to the mixture, and this was maintained under stirring for 3 h. When adding the TEOS to the ethanol and water mixture, the basic pH of the solution favored the condensation reaction, with the TEOS rapidly being transformed to Si-O-Si. After 3 h, a gel was obtained that was filtered and dried at 100 °C for 1 h to complete the condensation process. Finally, in order to obtain a porous material, the surfactant was removed at 550 °C for 3 h, obtaining a white powder. The calcination temperatures of the different surfactants were established by studying their degradation temperatures by means of a thermogravimetric analysis.

**Figure 2:**
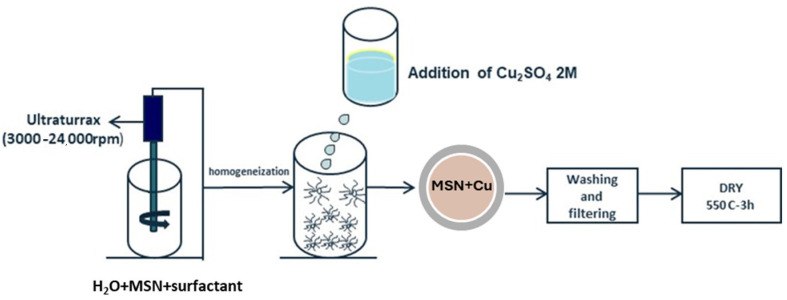
Synthesis route for the incorporation of Cu in preformed particles.

For the incorporation of Cu on the mesoporous structures, an impregnation process was employed. The MSNs were dispersed in water by using vigorous stirring in a Ultraturrax at 3000 rpm, using PVP (Polyvinyl pyrrolidone) as surfactant (SiO_2_/PVP ratio 1:0.1). Then, Cu_2_SO_4_ salt was dissolved in distillated water at a concentration of 1 wt.% of Cu with respect to the SiO_2_ content and the particles were maintained under stirring overnight. Finally, the samples were washed, filtered, and dried, obtaining Cu-modified silica mesoporous inorganic nanoparticles (Cu-MSN) as a blue powder. Two versions of Cu-MSNs were prepared: Cu-MSN-1, as synthesized without calcination, and Cu-MSN-2, in which the particles are subjected to a heat treatment at 550 °C for 3 h. This second version of the particles had a less intense blue, which could be due to the change in the oxidation state of Cu^2+^. The scheme for the synthesis was shown in Figure 2.

### 2.3. Physico-Chemical Characterization of Developed Cu-Modified MSNs

BET analyzer. Nitrogen (77 K) physisorption data was recorded on outgassed xerogel samples (vacuum at 150 °C for 10 h) with a Quantachrome Autosorb-iQ-MP (Quantachrome Instruments, Boynton Beach, FL, USA). The adsorption isotherm measurement using coated substrates was not feasible as the amount of sample deposited on the substrate was insufficient for the instrument sensitivity. The surface area values were obtained by the fittings of the N_2_ adsorption data to Brunauer–Emmett–Teller (BET) equation. The t-plot method was employed to assess the contribution of micropores and mesopores. The pore size distribution was modelled by density functional theory (DFT) as implemented in ASiQwin program of Quantachrome (ASiQwin V5.21, October 2010).

Laser Diffraction Spectroscopy (LDS). The particle size and the particle size distribution (PSD) of some of the developed particles were characterized by laser diffraction spectrometry (LDS). PSD was usually determined over a list of particle size ranges that covers nearly all sizes in the sample. Particle dispersity index (PdI) was used to evaluate the dispersion of PSD. LDS measurements were carried out in a Malvern Mastersizer 2000 instrument (Worcestershire, UK), using water as the dispersion media. A particle refractive index of 1.55 was considered for the analysis and 3 measurements were carried out for each sample.

Z-potential measurements. Z-potential measurements were performed to better understand the interactions between Cu-SMIN and microorganisms. The tests were carried out on a Malvern Zetasizer ZS equipment (Malvern Panalytical, Malvern, Worcestershire, UK) at 20 °C, using Cu-MSN-1 and Cu-MSN-2 dispersions in deionized water with a concentration of 1 mg/mL.

Scanning Electron Microscope (SEM). A Carl Zeiss SMT Ultra Gemini-II Field Emission Scanning Electron Microscope (FE-SEM) (Carl Zeiss SMT GmbH, Oberkochen, Germany) equipped with a Gemini column and an energy-dispersive X-ray fluorescence (EDS) system was employed. For the analysis of the particles, they were adhered to an aluminum support with a graphite tape. A voltage of 2 kV with an aperture of 30 µm was employed for the measurements.

Transmission Electron Microscopy (TEM). TEM images of 4096 × 4096 pixels were acquired with a JEOL JEM 2100 transmission electron microscope (JEOL, Akishima, Tokyo, Japan) operating at an acceleration voltage of 200 kV. For the analysis, the nanoparticles were suspended in distilled water and vortexed, and 5 µL was dispersed by drop-casting method onto an ultrathin carbon film on a Lacey carbon support film grid (TED PELLA, Inc., Redding, CA, USA) for TEM imaging.

Inductively Coupled Plasma Optical Emission Spectroscopy (ICP-OES). The copper content in Cu-MSN-1 and Cu-MSN-2 was quantified using a PerkinElmer Optima 8000 (PerkinElmer, Inc., Shelton, CT, USA). Approximately 10 mg of each sample was digested in 10 mL of 65% HNO_3_ under reflux at 120 °C for 2 h to ensure complete dissolution of silica and copper species. The resulting solutions were diluted to 50 mL with ultrapure water and analyzed at the Cu emission wavelength (324.754 nm). Calibration was performed using certified copper standards (0.1–10 mg/L), and results were expressed as weight percentage of copper relative to the total mass of the nanoparticles. Moreover, ICP-OES was also employed to determine the release of copper. Then, 100 mg of Cu-MSN-1 and Cu-MSN-2 were dispersed in 10 mL of deionized water, and the water was analyzed at time 0, 15, 30, 60, and 480 min. The released copper was expressed as weight percentage of copper relative to the total mass of copper in each particle, previously determined by ICP-OES after the digestion of the particles.

X-ray Photoelectron Spectroscopy (XPS). XPS measurements were performed in a Versaprobe III AD Physical Electronics (ULVAC) system (ULVAC‑PHI, Inc., Chigasaki, Kanagawa, Japan) with a monochromatic Al Kα radiation source (1486.7 eV). An initial analysis was carried out to determine the elements present (wide scan: step energy 0.2 eV, and pass energy 224 eV) and detailed analysis of the detected elements was carried out (detail scan: step energy 0.05 eV, pass energy 27 eV, and time per step 20 ms) with an electron exit angle of 45°. The spectrometer was previously calibrated with Ag (Ag 3d5/2, 368.26 eV). The spectra were adjusted using the CasaXPS 2.3.26 software, which models the contributions after a subtraction of the bottom (Shirley).

### 2.4. Evaluation of Antibacterial Activity

#### 2.4.1. Antibacterial Analysis

*S. aureus* ATCC 6538 (American Type Culture Collection) from Microbiologics (St. Cloud, MN, USA) was selected as the model strain for Gram-positive bacteria, and *E. coli* ATCC 8739 from Thermo Scientific (Waltham, MA, USA) was selected as the model strain for Gram-negative bacteria. The tests were conducted according to two experimental methods, namely, broth microdilution method to determine the Minimum Inhibitory Concentration (MIC) and Minimum Bactericidal Concentration (MBC), and the Time Killing Assay.

The MIC test determined the lowest concentration of an antimicrobial agent needed to inhibit the visible growth of bacteria. It provided a quantitative evaluation of microbial susceptibility to antibacterial agents [40,44,45]. A 0.5 McFarland turbidity standard was used to achieve the correct dilution by comparing turbidity values. At 600 nm, a 0.5 McFarland standard had an optical density between 0.08 and 0.1. This corresponded approximately to an *E. coli* suspension with a concentration of 1.5 × 10^8^ CFU/mL (Colony-Forming Units), which was then diluted to obtain the desired number of CFUs depending on the test to be performed. This standard was prepared by adding 99.5 mL of sulfuric acid (0.18 M) to 100 mL of purified water, followed by 0.5 mL of barium chloride (0.048 M) [46]. Bacterial strains were stored at −80 °C in cryovials containing porous spheres and grown overnight (ON) in liquid medium (Luria Bertani medium, LB, Sigma-Aldrich, MA, USA). The microorganisms were analyzed with a microdilution test in a 96-well cell culture plate (Eppendorf, Hamburg, Germany) to determine the MIC of the tested molecule. Each well had a final volume of 200 μL consisting of LB broth medium, 4 × 10^5^ CFU of bacterial suspension, and antimicrobial agent serially diluted 1:2 from the previous well to the next. Each experiment was performed in triplicate. Bacterial inoculum without antimicrobial agent was used as growth control, and sterile culture medium as a sterility control.

MBC test defined the minimum concentration of an antimicrobial agent required to kill a 99.9% of the bacterial population. This value corresponded to the concentration of antimicrobial substance at which no growth was observed on the agar plate (LB agar) after incubation at 37 °C ON [45].

The Time Killing Assay measured the bactericidal activity of a compound at a specific concentration and exposure time. The bacterial suspensions were used in the exponential growth phase, compared to the 0.5 McFarland turbidity standard, and then diluted to obtain a cell number equal to 4 × 10^4^ CFU. LB with bacteria (without antimicrobial agent) was used as a control to compare the bacterial growth of treated and untreated samples, and all tests were performed in triplicate. The bactericidal activity of the antimicrobial compound was calculated as percentage reduction in CFU number according to Equation (1):

(1) % Bactericidal activity = [1 − (CFU_E_/CFU_N_)] × 100

where CFU_E_ was the colonies’ number of the treated sample, and CFU_N_ was the mean colonies’ count of untreated samples [47].

#### 2.4.2. Statistical Analysis

Statistical analysis was performed using GraphPad Prism (Version 8.0.2, Boston, MA, USA). Data were presented as mean values of percentages ± standard deviation, and statistical differences were calculated by the Welch’s *t*-test. A two-tailed *p*-value less than 0.05 was considered statistically significant.

### 2.5. Evaluation of Virucidal Activity

#### 2.5.1. Virus Growth

SARS-CoV-2 variant omicron XBB.1.16.11 (hCoV-19/Italy/LAZ-DIBS-230829301/2023) was propagated in Vero cells cultured in MEM containing 2% (*w*/*v*) fetal bovine serum (Euroclone S.p.A., Pero, Milan, Italy). Influenza A (H1N1) pdm09 virus was propagated in Madin–Darby canine kidney (MDCK) cells cultured in Dulbecco’s Modified Eagle Medium (DMEM) containing 2% (*w*/*v*) fetal bovine serum (Euroclone S.p.A.), and 1 μg/mL trypsin-TPCK (Merck, Darmstadt, Germany). After 48–72 h from the infection, supernatants containing the released viral particles were collected and centrifuged at 600× *g* for 5 min. The viral titer was determined by plaque assay for SARS-CoV-2 and by 50% tissue culture infectious dose (TCID50) based on the method by Reed and Muench assay (1938) for A (H1N1) pdm09. Both viruses were isolated from nasopharyngeal swabs by the Defence Institute for Biomedical Sciences, Rome, Italy. Virus stocks were kept at −80 °C until use. MS2 bacteriophage (ATCC 5597-B1) was propagated using the host strain *E. coli* C-3000 (ATCC 15597) according to the instructions provided by ATCC. *E. coli* was cultured in Luria Bertani broth (LB) and then infected with the MS2 bacteriophage in the exponential to early stationary phase. After overnight incubation at 37 °C with shaking of 160 rpm, the phage culture was centrifuged at 4000× *g* for 10 min. The lysate was then filtered and stored at 4 °C.

#### 2.5.2. Virus Titration

SARS-CoV-2 plaque assay. Confluent Vero cell monolayers seeded in a 12-well plate were infected for 1 h at 37 °C with 0.2 mL aliquot of tenfold serial dilutions of SARS-CoV-2. Then, the inoculum was removed, and the cells were overlaid with MEM NEAA 1× (Gibco-Thermo Fisher Scientific S.p.A, Segrate, Italy), containing 1.5% Tragacanth (Sigma-Aldrich-Merck, Darmstadt, Germany), NaHCO_3_ 7% (Gibco- Thermo Fisher Scientific S.p.A, Segrate, Italy), L-glutamine 1× (Gibco-Thermo Fisher Scientific S.p.A, Segrate, Italy), 0.02 M Hepes (Euroclone S.p.A., Pero, Milan, Italy), DMSO (Sigma-Aldrich-Merck, Darmstadt, Germany ), and 2% FBS (Gibco-Thermo Fisher Scientific S.p.A, Segrate, Italy). Cells were incubated for 96 h in a CO_2_ incubator, and, then, the mixture was carefully removed, with plates washed with saline solution, and stained with 1% crystal violet for 10 min, and then plaque-forming units (PFU) were counted. The titer was expressed as plaque-forming units per milliliter (PFU/mL), which was the exact value of the viral infectivity. Virus propagation, exposition test, and plaque assays of SARS-CoV-2 need to be conducted in a bio-safety Level-3 facility according to WHO laboratory biosafety guidance.

Influenza A (H1N1) pdm09 virus TCID50 assay. Confluent monolayers of MDCK cells seeded in 96-well microtiter plate with a flat bottom were infected for 1 h at 37 °C with ten-fold serial dilutions of influenza A (H1N1) pdm09 virus tested in six replicates. Then, the inoculum was removed, and the cells were overlaid with D-MEM (Gibco) supplemented with 2% (*v*/*v*) FBS, 100 U/mL penicillin G, 100 μg/mL streptomycin, and trypsin at a final concentration of 1 μg/mL, and incubated for 48–72 h in a CO_2_ incubator. The plates were observed daily to monitor the cytopathic effects (CPEs). The endpoint was taken to be the highest dilution of the virus that produced CPE in 50% of the inoculated cells. Viral titers (50% tissue culture infective doses, TCID50/mL) were calculated using the Reed and Muench method.

MS2 bacteriophage plaque assay. The culture of *E. coli*, grown overnight at 37 °C with shaking of 160 rpm and OD600 = 0.3, was mixed with a soft agar (0.5%) solution and poured on LB agar plates to form a lawn. After the agar solidified, tenfold dilutions of MS2 bacteriophage were spotted on it, and the plates were incubated for 24 h at 37 °C. Then, the PFU were counted.

#### 2.5.3. Cytotoxicity Tests

The effect of Cu-MSNs on MDCK and Vero cell viability was evaluated by exposing confluent monolayers of cells to different concentrations (from 2.5 to 0.1 mg/mL) of the functionalized nanoparticles; the effects were observed after 24, 48, and 72 h by microscopic examination.

#### 2.5.4. Assessment of Virucidal Activity

The virucidal activity was assessed by comparing the replication competence of virions exposed to functionalized and unfunctionalized nanoparticles. The MSNs not functionalized were tested in order to confirm the virucidal inactivity and then used as positive control of viral infection. The titer of SARS-CoV-2, influenza A (H1N1) pdm09 virus, and MS2 bacteriophage was determined as described in Section 2.5.2. Aliquots of viral supernatant were added to Cu-MSNs suspension and incubated at 37 °C for different time points (15–30–60 min). The virucidal effects against SARS-CoV-2 and MS2 bacteriophage were assessed by the calculation of the plaque reduction ratio as follows: (100 − *N/N*_0_ × 100), where *N* is the PFU count of the viral suspension exposed to functionalized MSNs, and *N*_0_ is the PFU count of the positive control sample. The viral inhibition percentage was obtained from the means of the plaques from experiments performed in triplicate compared to positive control. Regarding influenza A (H1N1) pdm09 virus, the percentage of reduction was assessed by the calculation of the TCID50 reduction ratio: (T0 − T/T0) × 100, where T is the TCID50 value of the viral suspension exposed to functionalized MSNs and T0 is the TCID50 value of the positive control.

Statistical analysis was performed as explained in Section 2.4.2.

## 3. Results and Discussion

### 3.1. Synthesis and Physico-Chemical Characterization of Cu-Modified MSNs

MSNs were fabricated by the modified Stober’s method by using the soft template strategy. In the preparation of these materials, hydrolyzed silicate species condensate around a supramicellar assembly of cylindrical ionic surfactant micelles that act as the template, followed by the elimination of the template. The main parameters of the synthesized MSN, such as the particle and porous size, shape, and surface area, would depend on the synthesis parameters such as the reactant concentration, pH, and catalyst employed in the synthesis. An initial optimization of the synthesis conditions for the MSN nanoparticles was carried out, maintaining TEOS as the silica precursor and modifying the ratio of the other components including the surfactant type, catalyst type and concentration, and the presence of pore expanders.

To evaluate the effect of the surfactant type, two ionic surfactants, SDBS and CTAB, and two non-ionic surfactants, triblock copolymer Pluronic F127 and Tween 80, were used for the synthesis of mesoporous particles. These surfactants differ in size and their polarity. All of the synthesis was carried out at room temperature, maintaining the TEOS:EtOH:H_2_O:NH_4_OH:surfactant molar ratio of 1:20:50:0.5:0.1. Table 1 collects the different parameters employed during the preparation of the materials.

**Figure 3:**
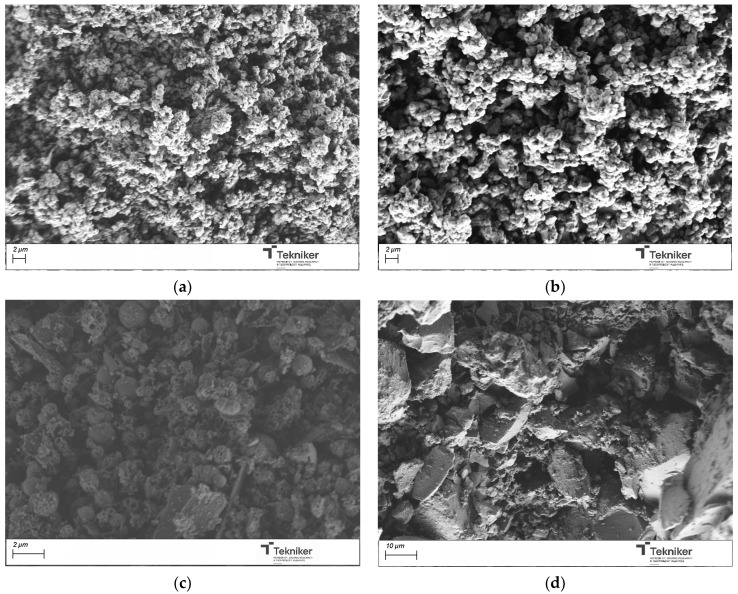
SEM image of the silica particles obtained by using different surfactants: (**a**) CTAB; (**b**) SDBS; (**c**) F127; and (**d**) Tween 80.

Once the samples were calcined at 550 °C for 3 h, the obtained powder materials were analyzed by SEM. The obtained images were shown in Figure 3. The results indicated that the use of F127 and T80 as a surfactant, at the analyzed conditions, did not lead to spherical shape particles, whereas the use of the ionic surfactants CTAB and SDBS leads to spherical particles of around 1000 nm in size, even if the observed particles seem to be aggregated. In the case of using SDBS, the aggregation degree of the particles was higher than for CTAB. The obtained particles were also subjected to an N_2_ adsorption analysis to study their morphological characteristics. Table 1 collected the main properties of the obtained materials. In the case of the supports synthesized with Pluronic F127 and SDBS, the material obtained did not have a high specific area, which is a necessary requirement to facilitate the incorporation of antimicrobial agents. The results indicated that CTAB yielded a homogeneous spherical particle size distribution with lower aggregation and with the highest specific area. This is well in accordance with the literature, as CTAB is a classic structure-directing agent for mesoporous silica [12,48,49,50], due to the strong electrostatic attraction between its positively charged micelles and the negatively charged silicate species present at high pH. This cooperative interaction stabilized the micelle–silica interface and promotes ordered silica condensation, leading to homogeneous spherical particles with a well-developed mesoporosity and high specific surface area.

**Figure 4:**
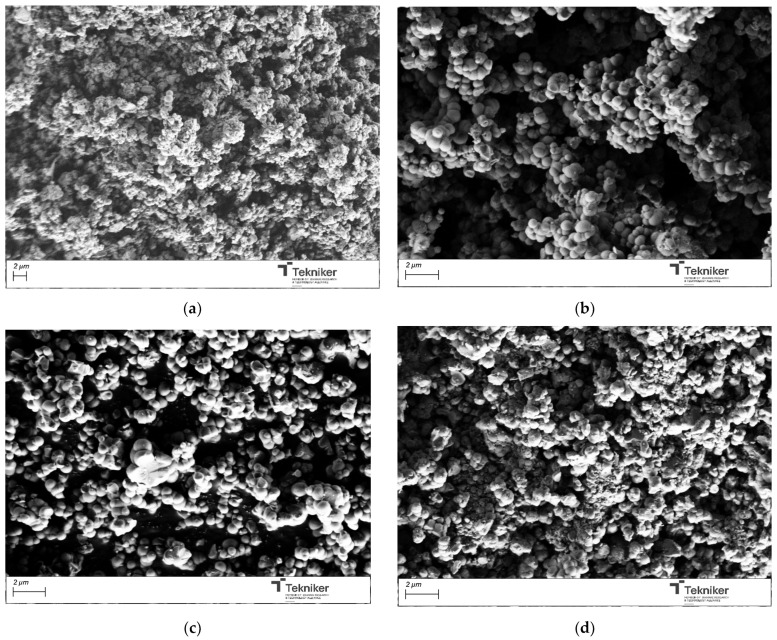
SEM image of the silica particles obtained by using different precursor:water ratios: (**a**) 1:50; (**b**) 1:100; (**c**) 1:500; and (**d**) 1:1000.

The effect of the precursor/water ratio on the proposed synthesis was also evaluated. A diluted concentration of the reactants and special silane were frequently adopted in the synthesis of MSNs with a small size (below 50 nm) to avoid the agglomeration of the synthesized nanoparticles, which inevitably resulted in low yields, a complicated post-treatment, and high costs. To evaluate the effect of the precursor/water ratio, the molar ratio of water in the mixture was increased. The different parameters employed during the preparation of the materials were collected in Table 1, along with the main characteristics of the synthesized materials. The size of the obtained particles was maintained in values around 1000 nm; thus, the precursor/water did not significantly affect the dimension of the particles (see Figure 4). However, this parameter affected the particles’ shape, as, by increasing the amount of H_2_O, more spherical NPs were obtained. Concerning the specific surface area, a significant increase in the BET value from 584 to 1131 m^2^/g was observed by changing the precursor:water ratio from 1:50 to 1:100 in the mixture. As the amount of water increased, the BET value remained relatively constant, and a slight aggregation of the particles appeared to occur. In addition, by increasing the water content in the reaction medium, the amount of nanoparticles obtained after the process was similar; thus, the optimum precursor:water value was selected as 1:100.

**Figure 5:**
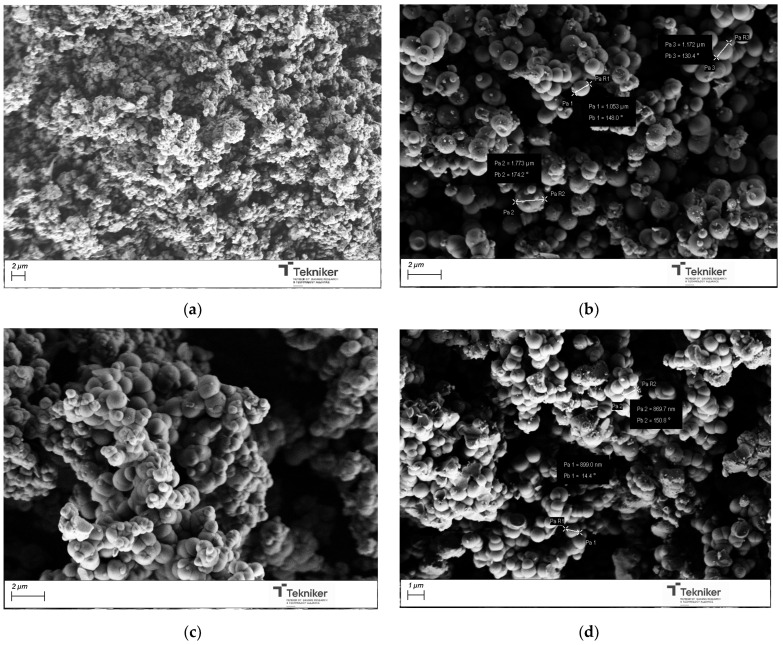
SEM image of the silica particles obtained by using different temperatures: (**a**) room temperature; (**b**) 60 °C; (**c**) 80 °C; and (**d**) 100 °C.

The synthesis temperature could also affect the main physico-chemical properties of the obtained particles. Different temperatures were selected for the synthesis, between room temperature to 80 °C, and the obtained particles were analyzed by SEM and N_2_ adsorption analysis, respectively. The images collected in Figure 5 indicate that increasing the reaction temperature resulted in a reduction in particle size from 1100 nm to 800 nm. However, the reaction temperature negatively affected the aggregation of the nanoparticles. Moreover, the BET data collected in Table 1 indicated that an increase in the reaction temperature reduced the BET value of the particles, which could be due to the increase in the rate of the reaction leading to the polycondensation of the silica monomers, resulting in a dense silica structure [51]; thus, a synthesis at room temperature was preferred.

**Figure 6:**
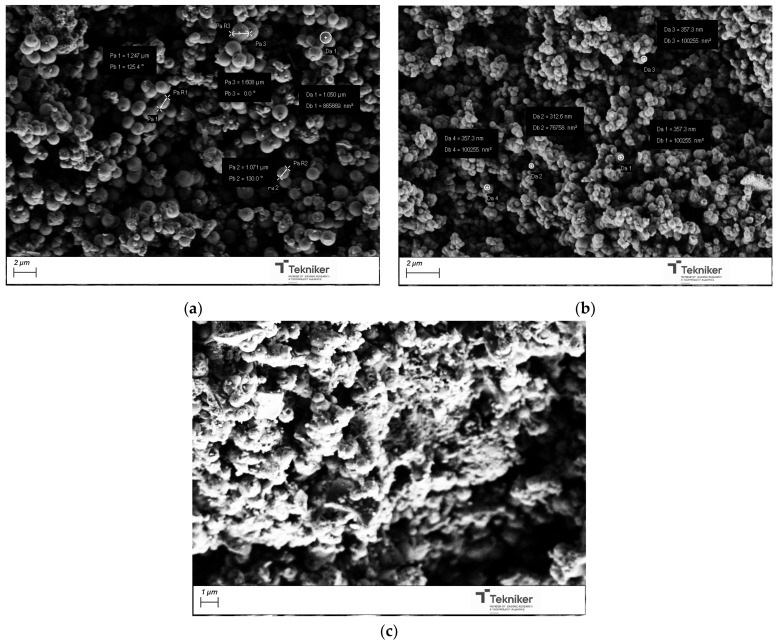
SEM image of the silica particles obtained by using different NH_4_OH catalyst concentrations: (**a**) NH_4_OH, (**b**) MEA, and (**c**) TEA.

The catalyst type and concentration could also have a big impact on the characteristics of the synthesized silica mesoporous particles. Three types of catalysts were employed: ammonium hydroxide (NH_4_OH, 28 wt.%), ethyl (methyl)amine (MEA), and triethylamine (TEA). The images of products obtained after their calcination, shown in Figure 6, indicated that, when using TEA as a catalyst, most parts of the silica were in bulk, instead of being in spherical particles. TEA could form some complexes, affecting the initial formation of the particles. When using MEA, spherical particles with sizes of around 350 nm could be obtained; however, the BET values of these particles were significantly reduced, as shown in Table 1.

**Figure 7:**
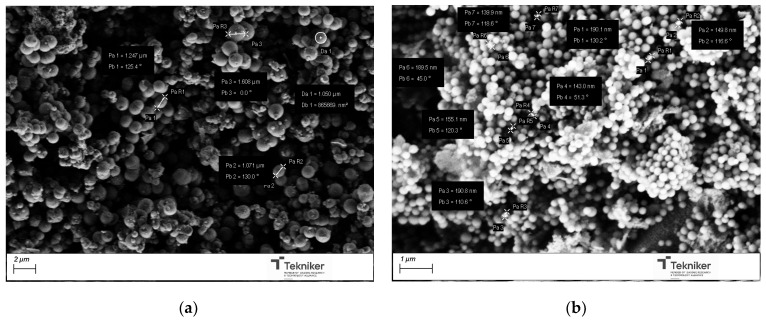
SEM image of the silica particles obtained by using different catalyst type: (**a**) NH_4_OH concentrated; and (**b**) NH_4_OH 2 M.

The effect of the base catalyst concentration used in the process was explored, using concentrated NH_4_OH solutions, as well as 1 M and 2 M solutions. As shown in Table 1 and Figure 7, the size of the obtained particles was significantly reduced with a change in the base catalyst concentration, from particles with diameters of around 1000 nm when using concentrated NH_4_OH to particles with diameters of 150 nm when NH_4_OH was used at a concentration of 2 M. The reduction in size can be achieved, maintaining a high specific surface area in the nanoparticles, so the use of NH_4_OH 2 M was selected for the next synthesis. It is worth mentioning that the reaction did not take place when the catalyst NH_4_OH 1 M was used, indicating the complex effect of the catalyst concentration on the kinetics of the reaction and, therefore, on the size of the generated nanoparticles.

Once we have analyzed the influence of the main parameters of the reaction, a full characterization of the pore sizes/volumes of the nanoparticles obtained by using the optimized reaction parameters was carried out. Thus, for the particles obtained by using the ratio TEOS:EtOH:H_2_O:NH_4_OH 2 M:CTAB—1:20:100:0.16:0.1, the parameters are as follows: particles with a size of between 140–250 nm, a BET area of 933 m^2^/g, and a pore volume and size of 1.172 cm^3^/g and 1.789 nm, respectively. In the development of mesoporous nanostructures, the use of a pore controller was common to allow the incorporation of antimicrobial agents and their release. In order to obtain particles with different pore sizes and evaluate the influence of these compounds on the size and properties of the synthesized MSN, a preliminary study was carried out using pore expanders with different structures or sizes. Table 2 collected the different parameters employed during the preparation of the materials, and also the main parameters of the resultant MSNs.

**Figure 8:**
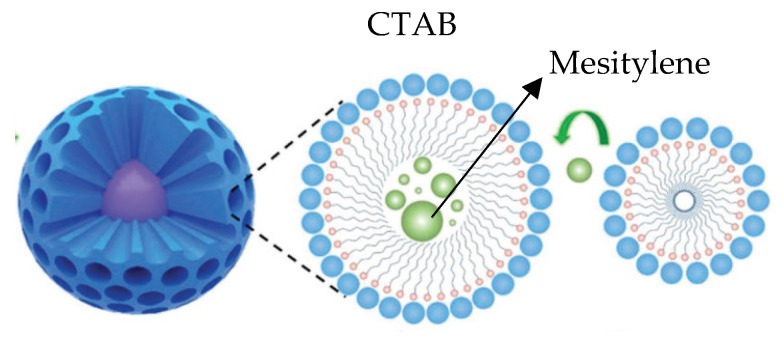
Possible mechanism of mesitylene as pore controller.

With the selected pore expander, the size of the pores in the nanoparticles changed from 1.3 to 6.0 nm, whereas the volume of the pores varied from 0.8 till 2.0 cm^3^/g. Mesitylene seemed to lead to better results in terms of increasing the pore size. Mesitylene could be introduced into the CTAB micelle during the emulsion formation to expand the diameter of the pores, as is suggested in Figure 8.

**Figure 9:**
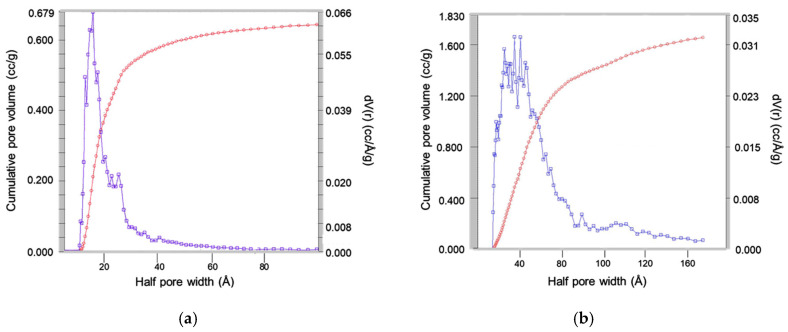
Analysis of pore size obtained by nitrogen isotherms of the silica particles obtained with the reactant ratio TEOS:EtOH:H_2_O:NH_4_OH 2 M:CTAB—1:20:100:0.16:0.1 using (**a**) no pore expander; and (**b**) 20 mL of mesitylene. Red curve represents the total accumulated pore volume and blue curve the differential pore volume distribution.

This increase in the pore size was clearly observed in the nitrogen isotherms obtained in the surface analyzer. Figure 9 showed the pore size distribution of the MSNs obtained with the reactant ratio TEOS:EtOH:H_2_O:NH_4_OH 2 M:CTAB—1:20:100:0.16:0.1 without any pore expander and the particles obtained using the same conditions but adding 20 mL of mesitylene in the synthesis procedure. This parameter, together with the specific area, was one of the most important parameters for these materials, as the pore size determines the release of the essential oil, which acted as an antibacterial agent.

**Figure 10:**
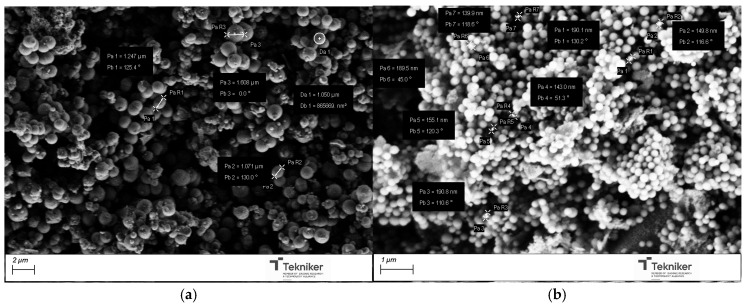
SEM image of the silica particles obtained with the reactant ratio TEOS:EtOH:H_2_O:NH_4_OH 2 M:CTAB—1:20:100:0.16:0.1 using (**a**) no pore expander; and (**b**) 20 mL of mesitylene.

The results also indicated that the presence of the pore expander also modified the final size of the particles, resulting in nanoparticles with sizes lower than 100 nm when 20 mL of mesitylene was incorporated in the synthesis process. It is worth mentioning that, for the MSN nanoparticles prepared with 20 mL of mesitylene, the synthesis procedure was also slightly different, including an additional step to homogenize the mesitylene in the mixture, where an emulsion was obtained by using an Ultraturrax IKA T25 (10,000 rpm, 15 min). Figure 10 shows the images of the MSNs obtained with the reactant ratio TEOS:EtOH:H_2_O:NH_4_OH 2 M:CTAB—1:20:100:0.16:0.1 without any pore expander and the particles obtained using the same conditions but adding 20 mL of mesitylene in the synthesis procedure. The reduction in the particle size was clearly observed, maintaining a good homogeneity in the particle shape and size distribution, and a lower aggregation in the synthesized nanoparticles. To verify that the obtained MSNs are in the targeted range of size, LDS was used to verify the size distribution of the MSNs obtained by using 20 mL of mesitylene as a pore expander, resulting in an average particle size d_50_ of 104 nm.

**Figure 11:**
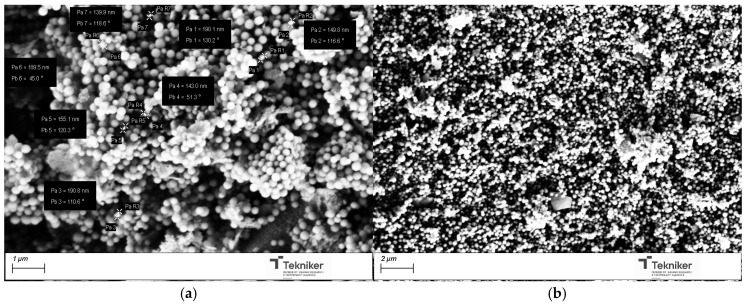
SEM image of the silica particles obtained with the reactant ratio TEOS:EtOH:H_2_O:NH_4_OH 2 M:CTAB—1:20:100:0.16:0.1 with 20 mL of mesitylene: (**a**) without Cu; and (**b**) with Cu.

**Figure 12:**
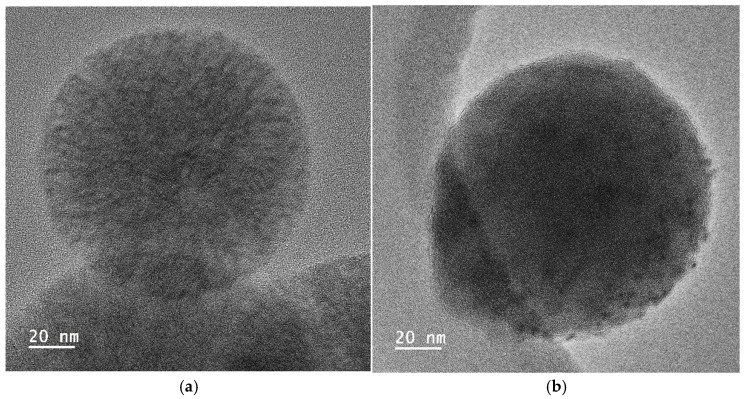
TEM micrographs of the silica particles obtained with the reactant ratio TEOS:EtOH:H_2_O:NH_4_OH 2 M:CTAB—1:20:100:0.16:0.1 with 20 mL of mesitylene: (**a**) without Cu; and (**b**) with Cu.

For the incorporation of the Cu salt in the preformed nanoparticles, the particles obtained by using the reactant ratio TEOS:EtOH:H_2_O:NH_4_OH 2 M:CTAB—1:20:100:0.16:0.1 with 20 mL of mesitylene were employed (average size = 104 nm, specific surface area = 900 m^2^/g, and pore size = 6 nm). After Cu incorporation, the SEM images of the nanoparticles, as shown in Figure 11, indicated that they had a similar shape, with no residues of salt observed. Moreover, the presence of Cu was clearly observed in the TEM micrographs corresponding to the modified nanoparticles, shown in Figure 12, where some clusters were observed on the surface of the nanoparticles. The presence of copper was analyzed by ICP-OES, with the presence of 1.5 and 1.3 wt.% copper being determined in Cu-MSN-1 and Cu-MSN-2, respectively.

**Figure 13:**
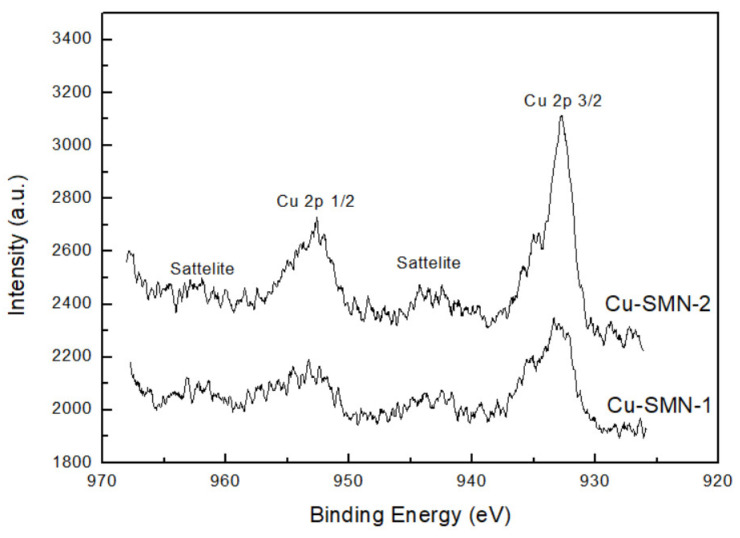
XPS spectra of Cu-MSN-1 and Cu-MSN-2.

Moreover, X-ray photoelectron spectroscopy analysis was used to investigate the structural composition and chemical states of Cu-SMN-1 and Cu-SMN-2, that is, the Cu-SMN as synthesized and after calcination. The XPS spectra were shown in Figure 13. The peaks at binding energies of 934.7 eV and 954.5 eV are attributed to Cu 2p_3/2_ and Cu 2p_1/2_, respectively. However, the peaks at binding energies of 943 eV and 962 eV in Cu-SMN are referred to as the satellite state of the Cu species. These satellite peaks are characteristic of the Cu^2+^ species, validating the presence of Cu and Cu^2+^ in both samples [52]. The quantitative analysis of both samples indicated that the content of Cu in the oxide state is 34 and 54%, and the content of the Cu^2+^ species is 67 and 46%, for Cu-SMN-1 and Cu-SMN-2, respectively. The values are quite similar, with the Cu in the oxide state in the Cu-SMN particle submitted to calcination being slightly higher.

### 3.2. Antimicrobial Characterization of Cu-Modified MSNs

#### 3.2.1. Antibacterial Characterization

The antimicrobial activity of nanoparticles against *E. coli* and *S. aureus* was evaluated using the MIC and MBC methods, as well as the killing test method. To determine the MIC and MBC, an MSN concentration in the first well of 2.5 mg/mL was diluted to 0.001 mg/mL. Then, 2.5 mg/mL was identified as the MIC value for both Cu-MSNs and both bacterial strains. For Cu-MSN-1, an MBC value of 2.5 mg/mL was also obtained for both bacteria. From the evaluation of MIC and MBC, Cu-MSN-1 was identified as the compound showing the highest bactericidal activity against both bacteria (an MBC value was obtained only for Cu-MSN-1).

**Figure 14:**
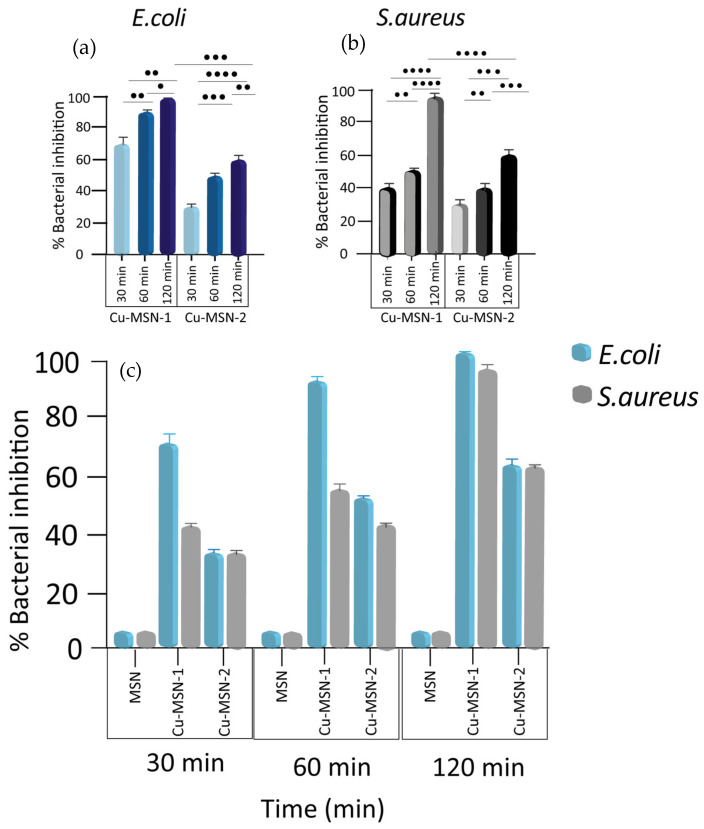
Nanoparticles’ antibacterial effect against *E. coli* and *S. aureus* over time. Bactericidal activity of Cu-MSN-1 and Cu-MSN-2 against (**a**) *E. coli* and (**b**) *S. aureus*. (**c**) Percentage of killing activity of MSN, Cu-MSN-1, and Cu-MSN-2 at a concentration of 2.5 mg/mL on *E. coli* and *S. aureus* over time (30, 60, and 120 min). Killing activity values were as calculated according to Equation (1) described in lines 271–279 and expressed as means ± the SD from three independent experiments. Statistical significance was indicated as • for values with *p* < 0.05; •• for values with *p* < 0.01; ••• for values with *p* < 0.001; and •••• for values with *p* < 0.0001.

The bactericidal test was also performed on both bacteria using the MSN preparations at a final concentration of 2.5 mg/mL for 30, 60, and 120 min. Complete killing activity was observed after 120 min of incubation only for Cu-MSN-1 on *E. coli*. Cu-MSN-2, instead, had only 60.4% of inhibition (*p* = 0.0009). On the other hand, the antibacterial activity of Cu-MSN-1 after 120 min against *S. aureus* was only 95.1% and the activity of Cu-MSN-2 reached 60.5% (*p* < 0.0001). The experiments were performed in triplicate, reporting the individual values obtained, their mean, and their standard deviation. These values indicate the good robustness of the data distributed close to the mean value. Both methods were also performed with MSNs without copper, which showed no antibacterial activity. The collected results were summarized in Figure 14.

A significative increase in the antibacterial activity of both Cu-MSNs against both bacteria was observed at all three times. Therefore, there were no significant differences at the different times (Figure 14a,b).

**Figure 15:**
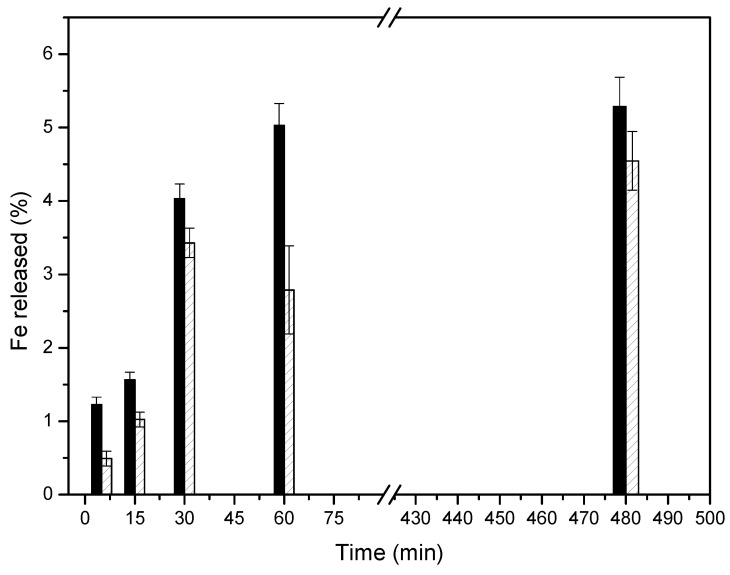
ICP-OES analysis of the Fe^2+^ released into the medium from (black) Cu-SMN-1 and (patterned) Cu-SMN-2.

The ζ-potential values of Cu-MSN-1 (−23.2 ± 0.1 mV) and Cu-MSN-2 (−25.3 ± 0.7 mV) indicate that both materials exhibit a similar negative surface charge. Calcination did not substantially modify the electrostatic profile of the external surface, and, therefore, electrostatic interactions with bacterial membranes cannot explain the differences in antibacterial activity observed between the two nanomaterials [53]. The slightly higher antimicrobial efficiency of Cu-MSN-1 could be due to the calcination process applied to obtain Cu-MSN-2. According to the XPS data, the calcination process could have converted copper salts (Cu^2+^) into less soluble oxide species, resulting in a slower release of Cu^2+^ into the environment [54]. To evaluate this statement, 100 mg of each nanoparticle was dispersed in 100 mL of deionized water and the release of Fe^2+^ into the water was monitored for 480 min by using ICP-OES analysis. The results were collected in Figure 15. It was observed that the leaching of Cu is slightly faster in Cu-SMIN-1, instead of Cu-SMIN-2, which could indicate the higher availability of these ions in Cu-SMIN-1.

#### 3.2.2. Virucidal Characterization

The effect of Cu-MSNs on cell viability was evaluated. The confluent monolayers of cells were exposed to different concentrations (2.5–0.1 mg/mL) of MSNs, and the effects were evaluated at 24, 48, and 72 h. A microscopic examination revealed no significant mortality in cells treated with the compounds at concentrations of up to 0.25 mg/mL. On the contrary, morphological alterations, the loss of cell viability, and the modification of the cell multiplication rate were observed at higher concentrations. Therefore, a first set of experiments to assess the virucidal activity of Cu-MSNs was performed, mixing viral suspensions (10^5^ pfu/mL) of SARS-CoV-2, influenza A (H1N1) pdm09 virus, and MS2 bacteriophage with functionalized and unfunctionalized nanoparticles at a final concentration of 0.25 mg/mL. The tests were carried out at 15, 30, and 60 min of exposure and a temperature of 37 °C.

**Figure 16:**
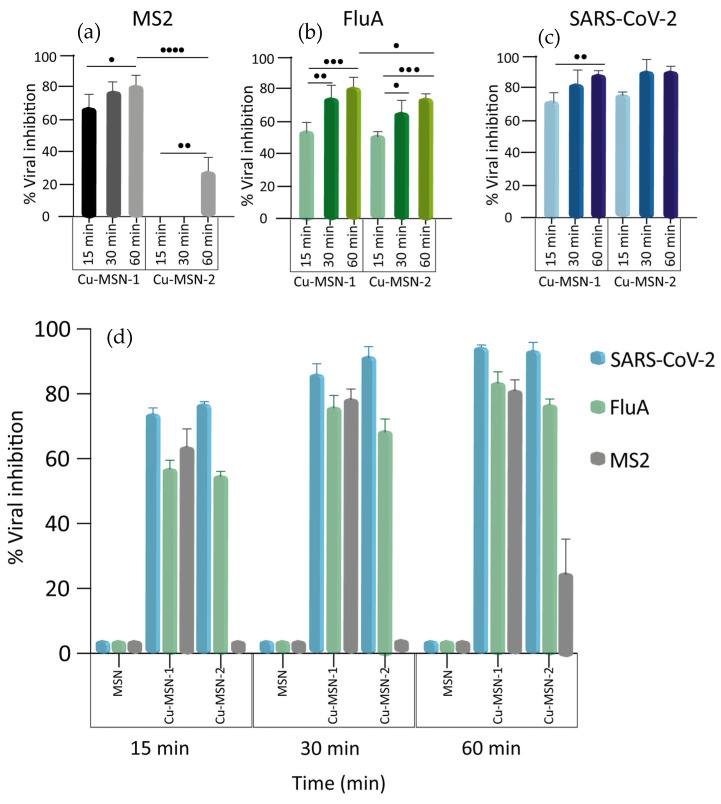
Nanoparticles’ virucidal effect against three viruses over time. Virucidal activity of Cu-MSN-1 and Cu-MSN-2 against MS2 bacteriophage (**a**), influenza A virus (**b**), and SARS-CoV-2 (**c**). (**d**) Overview of the inhibitory effects of MSN, Cu-MSN-1, and Cu-MSN-2 on the viruses. The viruses were incubated with the MSNs at a final concentration of 0.25 mg/mL for 15, 30, and 60 min and viral titers were calculated by plaque assay for MS2 and SARS-CoV-2 and TCID50 for influenza A. Viral titers, in the Y axis, were reported as percentage of inhibition. Values are expressed as means ± the SD from three experiments, each performed in triplicate (*n* = 9). Statistical significance was indicated as • for values with *p* < 0.05; •• for values with *p* < 0.01; ••• for values with *p* < 0.001; and •••• for values with *p* < 0.0001.

The results indicated that functionalized MSNs were able to inactivate the viruses with different efficiencies. As summarized in Figure 16, an inactivation ratio of 90% and 91.5% was observed with Cu-MSN-1 and 2, respectively, in SARS-CoV-2 at the highest time shift tested (*p* > 0.05), whereas influenza A was around 80% with Cu-MSN-1 and 75% with Cu-MSN-2 (*p* < 0.05). Regarding MS2, viral inhibition using Cu-MSN-1 reached 81.6%, whereas, with Cu-MSN-2, it was about 25% (*p* < 0.0001).

Interestingly, a significant increment over time of Cu-MSN-1’s virucidal activity was observed for all the viruses. The same results were obtained when treating the influenza A virus and the MS2 bacteriophage with Cu-MSN-2, whereas, for SARS-CoV-2, the degree of inhibition was high even at 15 min. For this reason, no significant differences were detected at the different time points (Figure 16a–c).

Furthermore, MS2 bacteriophage was also exposed at a final concentration of 2.5 mg/mL, obtaining an increment in the inhibition percentage as shown in Table 3.

The higher virucidal efficacy of Cu-MSN-1 compared to Cu-MSN-2 could be observed mainly in MS2 bacteriophage (*p*-value < 0.0001). It was attributed to the absence of a calcination step, which preserved the availability of surface Cu^2+^ ions. These features enhance both the direct interaction with viral particles and the generation of reactive oxygen species (ROS). This effect was particularly pronounced for non-enveloped viruses, that was MS2, whose protein capsids were more resistant and rely on immediate contact with Cu^2+^ or ROS for inactivation [55]. In contrast, enveloped viruses were more susceptible to oxidative damage of their lipid membranes, and, therefore, the reduced availability of Cu^2+^ in Cu-MSN-2 had a smaller impact on their inactivation.

## 4. Conclusions

The capacity of microorganisms to rapidly evolve resistance threatens a pillar of modern medicine, prompting a critical need for innovative approaches. In this study, copper-modified mesoporous silica nanoparticles (Cu-MSNs) were synthesized via a modified Stöber method using a soft-template approach. The influence of key synthesis parameters—including the surfactant type, precursor/water ratio, temperature, catalyst nature and concentration, and pore expanders—on the particle morphology and surface characteristics was systematically investigated. Among the surfactants tested, CTAB yielded homogeneous spherical nanoparticles with the highest specific surface area (up to 1131 m^2^/g). The optimization of the precursor/water ratio (1:100) enhanced sphericity and porosity, while synthesis at room temperature minimized aggregation. Using ammonium hydroxide 2 M as the catalyst produced nanoparticles of 140–250 nm with a high surface area (933 m^2^/g). Pore expansion using mesitylene increased the pore size from 1.3 to 6.0 nm and reduced the particle size to below 100 nm, yielding highly uniform and well-dispersed particles. Finally, copper incorporation by adding a water solution of Cu_2_SO_4_ salt was explored, and it was confirmed that this did not alter the morphology of the nanoparticles, as confirmed by SEM and TEM analyses, while TEM confirmed the homogeneous Cu distribution. Two versions of Cu-modified MSNs (Cu-MSN-1, without calcination, and Cu-MSN-2, with calcination) were obtained. The antimicrobial activities of these two Cu-MSN preparations were evaluated. From the evaluation of MIC and MBC, Cu-MSN-1 was identified as the compound with the highest bactericidal activity against *E. coli* and *S. aureus*. Both nanoparticles were also assessed using time-assays, showing that only Cu-MSN-1 achieved complete bactericidal activity after 2 h of exposure to *E. coli*. Both particles had a similar negative surface charge, so the higher activity of Cu-MSN-1 could be ascribed to the presence of more soluble Cu^2+^ species, as was observed by XPS and ICP-OES.

Finally, the ability of Cu-MSNs to inactivate enveloped RNA viruses such as the influenza A virus (pH1N1) and the severe acute respiratory syndrome coronavirus 2 (SARS-CoV-2), as well as MS2, used as a reference model for non-enveloped viruses, was evaluated. In vitro test results demonstrated a significant decrease, approximately 90%, in the viability and infectivity of SARS-CoV-2 after exposure to both Cu-MSNs at the highest time shift tested; a lower reduction in infectivity, approximately from 75% to 80%, was observed in flu A. MS2 bacteriophage was inactivated by almost 80% when incubated with Cu-MSN-1 at a final concentration of 0.25 mg/mL and by 30% with Cu-MSN-2. A tenfold increment in the concentration of both the MSNs investigated led to an increment in the viral inhibition percentage, reaching 97% for Cu-MSN-1 and 70% for Cu-MSN-2. These results demonstrate that Cu-modified mesoporous silica nanoparticles combine the high surface area and tunable porosity with significant antibacterial and antiviral efficacy, making them promising candidates for applications in antimicrobial coatings on high-touch surfaces in healthcare settings, food packaging, and water treatment membranes, as well as antiviral textiles for personal protective equipment. Compared to silver-based MSN systems, copper provides a similar broad-spectrum antimicrobial activity at a fraction of the cost and with lower environmental and toxicological concerns. Unlike antibiotic-loaded MSNs, copper-functionalized MSNs act through multiple mechanisms, including membrane disruption and ROS generation, reducing the risk of resistance development. Future work should explore the long-term stability, reusability, and integration into final application.

## Figures and Tables

**Table 1:** Synthesis conditions for the mesoporous silica and main properties of the materials.

Ratio	T (°C)	pH	Size (nm)	BET (m^2^/g)
TEOS:EtOH:H_2_O:NH_4_OH:CTAB—1:20:50:0.5:0.1	Room	12.27	1000	584.2
TEOS:EtOH:H_2_O:NH_4_OH:SDBS—1:20:50:0.5:0.1	Room	12.03	1000	3.7
TEOS:EtOH:H_2_O:NH_4_OH:F127—1:20:50:0.5:0.1	Room	11.98	--	446.2
TEOS:EtOH:H_2_O:NH_4_OH:Tween80—1:20:50:0.5:0.1	Room	11.38	--	-
TEOS:EtOH:H_2_O:NH_4_OH:CTAB—1:20:50:0.5:0.1	Room	12.27	1000	584.2
TEOS:EtOH:H_2_O:NH_4_OH:CTAB—1:20:100:0.5:0.1	Room	12.23	1000	1131.0
TEOS:EtOH:H_2_O:NH_4_OH:CTAB—1:20:200:0.5:0.1	Room	12.01	1000	1064.0
TEOS:EtOH:H_2_O:NH_4_OH:CTAB—1:20:1000:0.5:0.1	Room	12.15	1000	938.5
TEOS:EtOH:H_2_O:NH_4_OH:CTAB—1:20:100:0.5:0.1	Room	12.23	1100	1131.0
TEOS:EtOH:H_2_O:NH_4_OH:CTAB—1:20:100:0.5:0.1	60	11.99	1000	632.0
TEOS:EtOH:H_2_O:NH_4_OH:CTAB—1:20:100:0.5:0.1	80	12.09	800	630.0
TEOS:EtOH:H_2_O:NH_4_OH:CTAB—1:20:100:0.5:0.1	100	12.15	800	290.0
TEOS:EtOH:H_2_O:NH_4_OH:CTAB—1:20:100:0.5:0.1	Room		1100	1131.0
TEOS:EtOH:H_2_O:MEA:CTAB—1:20:100:0.5:0.1	Room		350	362.0
TEOS:EtOH:H_2_O:TEA:CTAB—1:20:100:0.5:0.1	Room		800	n.d
TEOS:EtOH:H_2_O:NH_4_OHconc:CTAB—1:20:100:0.5:0.1	Room		1100	1131.0
TEOS:EtOH:H_2_O:NH_4_OH 2 M:CTAB—1:20:100:0.16:0.1	Room		140–250	933.0
TEOS:EtOH:H_2_O:NH_4_OH 1 M:CTAB—1:20:100:0.08:0.1	Room		No reaction	-

**Table 2:** Synthesis conditions for the mesoporous silica using different type of pore expanders and main properties of the materials.

Ratio	Pore Controller	Size (nm)	BET (m^2^/g)	Pore Volume (cm^3^/g)	Pore Size (nm)
TEOS:EtOH:H_2_O:NH_4_OH 2 M:CTAB—1:20:100:0.16:0.1	-	140–250	933.0	1.172	1.789
	Heptane—20 mL	140–250	808.8	0.910	2.500
	Heptane—40 mL	140–250	1077.5	1.471	1.589
	Dodecane—40 mL	140–250	1003.7	0.825	1.291
	Cyclohexane—40 mL	140–250	1005.6	2.033	1.657
	Mesitylene—20 mL	90–100	910.0	0.997	6.020

**Table 3:** Virucidal effect of Cu-MSN-1 and Cu-MSN-2 against MS2, over time. The virus was treated with MSNs at a concentration of 2.5 mg/mL, and viral titer inhibition is expressed as viral inhibition percentage obtained from the means of the plaques from experiments performed in triplicate.

Sample	Incubation Time (min)	Viral Inhibition (%)
		MS2
Cu-MSN-1	15	69.2
	30	83.9
	60	97.2
Cu-MSN-2	15	7.69
	30	26.9
	60	70.3

Values are expressed as viral inhibition percentage obtained from the means of the plaques from experiments performed in triplicate.

## Data Availability

The data presented in this study are available upon request from the corresponding author.
